# Fusion of Embedded Vision and Intelligent Algorithms for Non-Contact Deformation Monitoring

**DOI:** 10.3390/s26113338

**Published:** 2026-05-25

**Authors:** Mei Dong, Xinyu Liu, Hui Hu, Eisha Zahra, Kuihua Wang

**Affiliations:** 1 Research Center of Coastal and Urban Geotechnical Engineering, Zhejiang University, Hangzhou 310058, China; mdong@zju.edu.cn (M.D.); 22512166@zju.edu.cn (X.L.); 22512318@zju.edu.cn (E.Z.); 2 Zhejiang Key Laboratory of the Development and Utilization of Underground Space, Zhejiang University, Hangzhou 310058, China; 3 Hangzhou Ruhr IoT Technology Co., Ltd., Hangzhou 310023, China; huhui@ruhrtec.cn

**Keywords:** intelligent vision, deformation monitoring, subpixel localization, bridge deflection, sluice structure

## Abstract

With the increasing demand for reliable structural safety assessment in service, high-precision, non-contact, and long-term deformation monitoring has become increasingly urgent for large civil engineering structures. To address this need, this study proposes and validates a system-level non-contact monitoring framework that integrates an embedded vision-based deformation sensor with intelligent algorithms. Rather than treating individual techniques as isolated components, the proposed framework integrates high-precision optical imaging, subpixel localization, and intelligent image processing into a unified monitoring workflow. By continuously imaging and tracking targets on the structural surface, high-precision acquisition of two-dimensional dynamic displacements is achieved. To address issues such as image jitter, environmental disturbances, and camera-induced vibrations under long-distance imaging conditions, a hybrid algorithm based on signal processing and image correction is introduced to effectively compensate and filter the monitoring data, thereby significantly improving the stability and accuracy of deflection measurements. In engineering applications, a girder bridge and an integral open-box sluice structure were selected as monitoring objects, and field experiments were conducted over multiple periods under different working conditions. The results indicate that the proposed system can stably capture small structural displacements, achieving sub-millimeter measurement accuracy. The findings verify the feasibility and reliability of the proposed intelligent vision-based deformation monitoring technology in complex engineering environments, and provide a new technical approach for structural safety assessment and operational monitoring of infrastructure such as bridges and hydraulic structures.

## 1. Introduction

Structural Health Monitoring (SHM) is a critical technique to ensure the safe operation of major infrastructures such as bridges and hydraulic structures [1]. During service, structures are subjected to various influences including traffic loads, hydrodynamic forces and ambient temperature variations; their deformation and displacement characteristics directly reflect their stress state and functional performance [2]. Therefore, obtaining high-precision, continuous and reliable displacement measurements is of significant importance for structural safety assessment, deterioration detection and maintenance decision-making.

Conventional displacement monitoring primarily relies on contact sensors such as displacement transducers, strain gauges and accelerometers [3,4,5]. While these methods provide high local measurement accuracy, they often face deployment difficulties, high maintenance costs and limitations in measurement coverage for large-scale structures or complex environments. In recent years, Global Navigation Satellite Systems (GNSS) and vision-based non-contact monitoring approaches have attracted increasing attention [6]. Vision-based techniques offer flexible deployment, rich information content and suitability for multi-point synchronous measurement, making them a promising approach for structural monitoring applications [7,8].

However, the direct application of vision-based methods to long-term field monitoring presents several challenges. Under long-distance imaging conditions, limitations in camera resolution, image plane jitter and thermal disturbance effects can substantially degrade displacement accuracy [9]. Moreover, environmental factors such as illumination changes, camera platform micro-motions and temperature variations can introduce low-frequency spurious signals, reducing data stability and reliability [10,11,12,13]. In addition, most existing studies focus on short-term or controlled experiments and discussions of long-term performance under cyclic loading in real engineering scenarios remain limited.

Motivated by these issues, this study presents the system-level co-design and field validation of an embedded vision-based deformation sensor and intelligent algorithms for long-term structural monitoring. Specifically, the proposed framework integrates high-precision optical imaging, subpixel localization, and interference-robust signal processing into a unified monitoring chain, rather than treating these techniques as isolated components. This integration enables stable displacement acquisition under long-distance imaging conditions and improves the robustness of the monitoring process in complex outdoor environments. To evaluate the engineering applicability of the proposed approach, field experiments were conducted on a girder bridge and an integral open-box sluice, and the structural displacement responses under traffic loading and tidal water-level variations were systematically analyzed. The results indicate that the method achieves sub-millimeter displacement detection in long-term field monitoring and demonstrates good robustness and practical engineering applicability.

## 2. Related Work

Vision-based methods for displacement and deformation monitoring have been extensively investigated in the civil engineering literature in recent years.

Single-point visual monitoring is among the earliest and most widely studied approaches in vision-based SHM. In this paradigm, displacement time series are inferred by tracking artificial targets or naturally occurring feature points on the structural surface. Aliansyah et al. implemented a single-camera, dual-view cooperative monitoring system that combines a forward-looking camera with a specially arranged marker array, enabling synchronous sub-millimeter measurements at multiple discrete points within a single lens field of view [14]. Peng proposed a single-camera method for bridge damage detection that extracts a transverse displacement influence ratio (DTIR) between girders to achieve high-sensitivity damage identification [15]. These methods are simple to implement and computationally efficient; however, they remain sensitive to occlusion, illumination changes and camera pose perturbations, which limits their suitability for long-term monitoring in complex field conditions.

To increase spatial coverage, multi-point and multi-view visual monitoring approaches have been developed. By deploying multiple targets or cameras, these methods enable observation of the overall deformation pattern of a structure. Micozzi proposed a system capable of real-time extraction of displacements at multiple deck cross-sections, emphasizing ease of replication and cost-effectiveness [16]. Chen developed a large-scale multi-point dynamic displacement monitoring system based on a dual-head camera chain (DHC). By physically linking optical paths and applying motion-error compensation models, the system addresses the trade-off between measurement range and accuracy in large-scale visual monitoring [17]. Lydon presented a multi-camera mobile vision system for bridge anomaly detection that employs four synchronized cameras and a camera rotation strategy to achieve low-cost, multi-point acquisition and full-field displacement reconstruction [18]. Although multi-view approaches can reconstruct more complete deformation fields, they impose higher demands on system calibration, synchronization and data fusion, and making them more challenging to deploy in the field.

For long-distance and outdoor applications, several studies have explored long-focal-length optics, image enhancement, and temperature compensation techniques to mitigate resolution loss and thermal effects. Researchers have also investigated various interference-robust algorithms including filtering, time-frequency analysis, and machine learning approaches, to address artifacts induced by illumination changes and platform motion. For example, Dong demonstrated non-contact displacement monitoring over ultra-long distances (600–1350 m) on three landmark suspension bridges with main spans exceeding 1000 m, verifying the feasibility and accuracy of such methods for very long-range measurements [19]. Liu proposed a subpixel matching algorithm based on a dual-precision gradient method that uses dual interpolation centers and weighted fusion to improve DIC accuracy and noise robustness [20]. Shanshan introduced a deep learning framework integrating video super-resolution and unsupervised homography estimation to address low image resolution and camera motion in large-scale monitoring, showing particular promise under low-illumination conditions [21]. Nevertheless, most of these advances focus on individual technical components rather than on system-level integration and extended field validation.

In recent years, data-driven approaches have also gained increasing attention in structural engineering applications. These methods leverage machine learning and hybrid data–model frameworks to improve the efficiency and accuracy of structural response prediction, fatigue assessment, and damage evolution analysis. Recent studies have increasingly explored data-driven and intelligent diagnosis methods for structural health monitoring applications. For example, interpretable machine learning models such as random forest surrogates have been applied for rapid stress intensity factor prediction and fatigue life evaluation of steel bridge components [22]. Other studies have investigated data-driven or hybrid data–model-driven strategies for fatigue stress response prediction and crack propagation analysis, demonstrating strong potential for capturing complex structural behaviors under varying operational conditions [23,24].

In summary, although existing vision-based research has made considerable progress in algorithmic development and accuracy improvement, gaps remain in long-term stability, interference resistance, and the depth of engineering validation under complex field conditions. In parallel, recent data-driven studies have demonstrated the growing potential of machine-learning-based and data–model-driven approaches for structural response prediction and damage assessment, yet these works still largely focus on isolated algorithmic performance rather than integrated sensing and interpretation. Building on prior work, the present study adopts a system-level perspective that combines hardware engineering and algorithmic co-design to propose and validate an intelligent vision-based deformation sensing solution intended to address these practical deficiencies.

## 3. Methodology

The methodology of this study is centered on vision-based deformation sensing technology. In the research design, the structure is taken as the monitoring object, and a non-contact monitoring scheme based on visual perception is established, including the specification of equipment selection, measurement point layout, and experimental conditions. For data acquisition, a self-developed vision-based deformation sensor is employed, integrating high-precision optical imaging and subpixel localization techniques to achieve real-time measurement of structural displacement. To mitigate environmental interference, a hybrid approach combining Hilbert–Huang transform (HHT) filtering and surface-fitting-based subpixel algorithms is introduced to enhance measurement accuracy and stability. For data processing and analysis, displacement signals are analyzed through outlier removal, moving average analysis, and threshold-based peak detection (μ + 3σ), enabling trend extraction, stability evaluation, and transient event identification. In addition, statistical indicators such as mean displacement and standard deviation are computed, forming a complete framework from signal acquisition to structural behavior interpretation.

### 3.1. Data Acquisition and Vision-Based Monitoring Method

**Figure 1:**
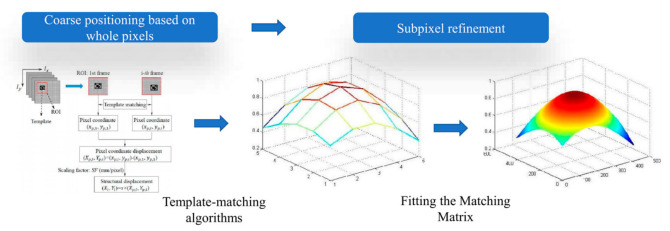
Basic Principles of Visual Deformation Monitoring Technology.

The vision-based deformation sensor is an intelligent sensing device that integrates video acquisition, image processing, and data transmission functions, and is developed based on an ARM + Linux architecture. As shown in Figure 1, the fundamental principle of vision-based deformation monitoring is to continuously capture the dynamic displacement of a structure over a given time period using a camera equipped with a high-precision optical lens. The acquired image sequence is then processed by an embedded image processing unit, and subpixel techniques are applied to obtain high-accuracy structural vibration displacements. Through coordinate transformation, the pixel-level displacement is converted into displacement time-history data expressed in physical distance units, enabling the measurement of bidirectional displacements in both horizontal and vertical directions.

The vision-based deformation sensor adopts a modular design in which the hardware system and software algorithms operate in coordination, allowing stable operation under complex environmental conditions and continuous output of structural deformation data. With the support of a high-resolution optical imaging system and advanced image processing algorithms, the sensor is capable of accurately capturing small structural displacements, thereby providing a reliable data foundation for subsequent structural deformation analysis and health monitoring.

At the same time, the proposed vision-based deformation sensor is implemented on an ARM-based edge architecture and supports local data storage. During temporary network interruption, the acquired monitoring data are first cached locally and then transmitted after the network connection is restored. The uploaded data are validated at the monitoring platform using time stamps and sequence continuity to ensure consistency between the local and online records, thereby preventing data loss or duplication.

If the interruption persists for an extended period, the condition is treated as a system anomaly and may indicate either sensor malfunction or site-level issues. In practical field deployment, such cases require manual inspection and on-site troubleshooting. This local-storage and timestamp-based recovery strategy ensures reliable long-duration data transmission for structural monitoring applications.

### 3.2. Image Processing and Measurement Accuracy Enhancement Methods

To further improve monitoring accuracy, targeted technical enhancements are introduced in the image processing stage. The proposed method follows a sequential processing workflow, including image-based target tracking, signal denoising, subpixel localization, and displacement reconstruction. First, image sequences acquired by the vision-based sensor are used to track the target position frame by frame, forming the raw displacement signal. Then, a noise-assisted Hilbert–Huang Transform (HHT) scheme is adopted to suppress low-frequency interference caused by camera vibration and environmental disturbances. Specifically, the deflection signal is superimposed with predefined Gaussian white noise, and the ensemble empirical mode decomposition (EEMD) process is applied to decompose the signal into a set of intrinsic mode functions (IMFs) and a residual term.

The amplitude of the added Gaussian white noise is determined based on commonly adopted practices in the literature, where it is typically selected as a proportion of the standard deviation of the original signal [25]. In this study, a value of 0.2 times the standard deviation is adopted, and preliminary tests were conducted to verify its effectiveness. This choice provides a balance between mitigating mode mixing and avoiding excessive residual noise. A smaller noise amplitude may lead to insufficient separation of intrinsic modes, while an excessively large amplitude may introduce additional background noise after reconstruction. To reduce boundary effects, standard signal extension techniques are applied at both ends of the time series.

After decomposition, the low-frequency components associated with inter-frame jitter and temperature-induced image distortion are subsequently filtered out, while the meaningful displacement trend is retained. This procedure effectively improves the robustness of the measured deflection signal under long-distance imaging conditions [26,27].

Meanwhile, a surface-fitting-based subpixel localization method is employed to refine the target position. In this step, the intensity distribution around the target is approximated by a local fitting surface, and the subpixel target center is determined by solving the extremum of the fitted surface. By reducing the localization error to better than 0.1 pixels, this method advances pixel-level resolution to the sub-millimeter scale and significantly improves displacement measurement accuracy [28,29,30].

Finally, to support engineering applications, an intelligent algorithm development platform is established to integrate conventional vision-processing modules and newly developed algorithms in a modular framework, while maintaining compatibility with commonly used deep learning frameworks. In addition, an intelligent-assisted screening step is introduced during preprocessing to support anomaly identification and improve data quality control. Thereby improving the reliability and interpretability of early warning results. The overall workflow of noise-assisted filtering, subpixel refinement, and intelligent anomaly recognition can be summarized as a sequential processing pipeline from raw image acquisition to displacement estimation and risk assessment.

### 3.3. Data Analysis Methods

**Figure 2:**
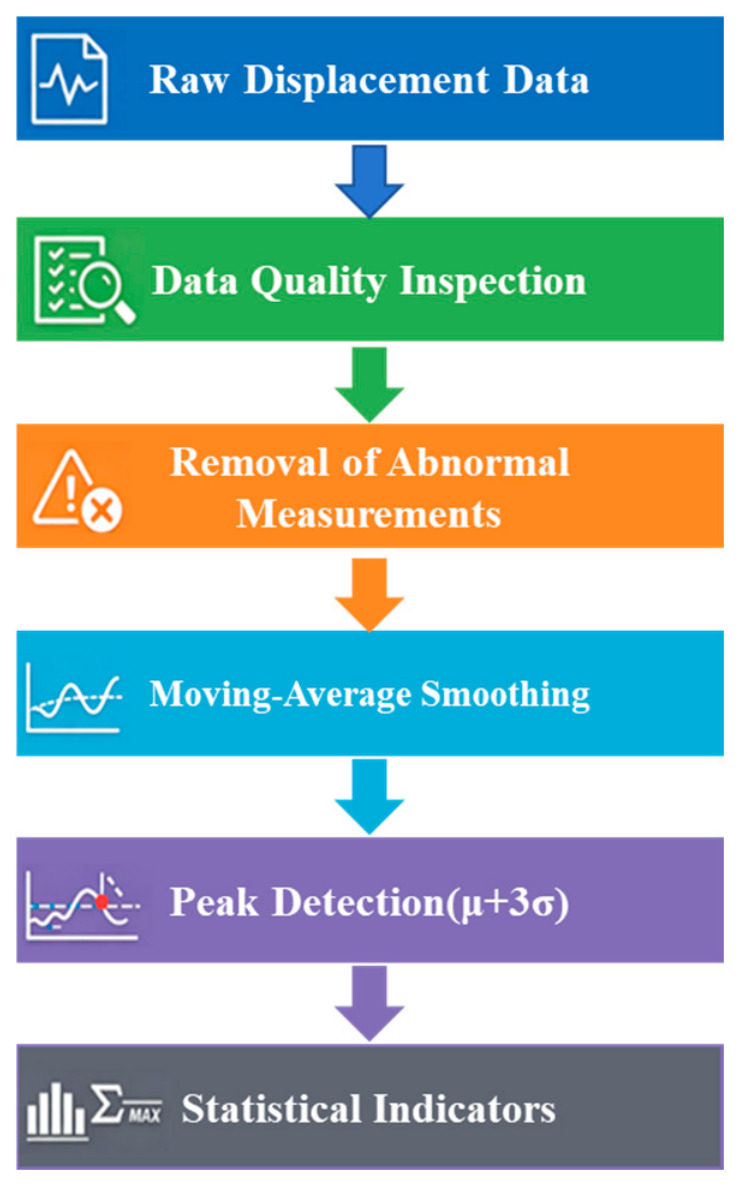
Data Analysis Workflow Diagram.

The displacement data used in this study are structural displacement time series obtained from a vision-based monitoring system. The analysis process is shown in Figure 2. The dataset consists of timestamped two-dimensional displacement measurements, which are used to characterize the dynamic response of the structure during operation.

Prior to statistical analysis, the raw data is subjected to quality inspection and preprocessing. Preliminary time-series visualization and data integrity checks reveal a short period of unstable measurements within the dataset. These abnormal measurements are primarily attributed to transient disturbances during the visual tracking process, such as temporary occlusion or image recognition errors. To ensure the reliability of subsequent analysis, the unstable segment was removed, while the remaining stable observations were retained for further analysis.

In addition, baseline drift is compensated using a reference-based calibration approach. A fixed reference target is installed in a non-deforming region to provide a stable displacement baseline. Since the theoretical displacement of the reference target is zero, any measured variation in its displacement is interpreted as system drift caused by environmental disturbances or camera motion. This drift component is subsequently removed from the monitoring results to obtain the corrected displacement time series.

After preprocessing, time-series visualization is employed for preliminary analysis of the displacement signals to evaluate the overall operational condition of the monitoring system during the observation period. To reduce the influence of random noise on short-term fluctuations and to better reveal displacement trends, a moving average method with a fixed time window is applied. The calculation is expressed as

$${\bar{x}}_{t}=\frac{1}{n}\sum_{i=0}^{n-1}x_{t-i}$$

where ${\bar{x}}_{t}$ denotes the moving average at time t, $x_{t-i}$ represents the displacement at the i-th time step within the window, and n is the window size. A rolling mean with a window size of 6 samples (approximately 1 h) is applied to smooth the displacement signal while preserving its overall trend. The selected window size represents a balance between noise reduction and trend preservation. A shorter window would retain more high-frequency fluctuations, whereas a larger window could excessively smooth the signal and obscure transient displacement responses. This method smooths high-frequency noise while preserving the overall variation characteristics of the signal [31].

To identify potential transient displacement responses during monitoring, a statistical peak detection method is further applied to the displacement series. Specifically, a threshold criterion of *μ* + 3*σ* is adopted for detecting abnormal displacement events, expressed as

T=μ+3σ

where T is the peak detection threshold, μ is the mean of the displacement series, and σ is the standard deviation of the displacement signal. A displacement value is identified as a potential peak event when

$$\left|x_{t}\right|>T$$

This approach effectively detects rare extreme responses in the time series while maintaining robustness to normal structural displacement variations [32].

To further quantify the statistical characteristics of structural displacement, several statistical indicators are computed, including mean displacement, standard deviation, maximum displacement, and the number of detected peak events. These metrics provide a quantitative description of the overall deformation level and its variability during the monitoring period, and offer a basis for identifying transient structural responses induced by external loads such as vehicle passages.

## 4. Vision-Based Monitoring Sensor

**Figure 3:**
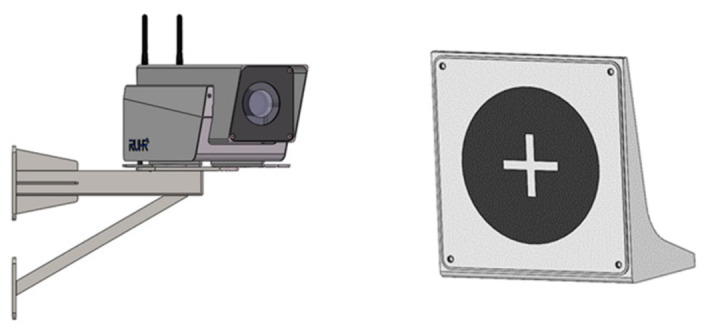
Appearance of the visual inspection sensor.

The vision-based deformation monitoring system adopted in this study enables long-distance (1–500 m), high-precision (sub-millimeter), non-contact displacement measurement, as shown in Figure 3. According to laboratory calibration results, the system achieves a measurement accuracy on the order of 0.2 mm at an observation distance of approximately 50 m. As the observation distance increases, the measurement accuracy exhibits a predictable degradation due to reduced effective pixel occupancy and an increased scale factor. Under identical imaging conditions with a 10 mm reference displacement, the accuracy is approximately 0.2 mm at 50 m, 1 mm at 200 m, and 2 mm at 400 m; however, within a range of several hundred meters, it still satisfies the accuracy requirements for deformation monitoring of engineering structures.

The accuracy of vision-based measurement largely depends on the design and arrangement of monitoring targets [10,33]. To ensure sufficient image resolution, the target should occupy approximately one-fifth to one-half of the camera’s field of view. This placement is an empirical guideline based on imaging geometry and preliminary tests. Targets positioned near the edges of the field of view tend to exhibit larger displacement fluctuations due to off-axis viewing effects; therefore, placing the target in the central region improves measurement stability. The target size can be selected according to the monitoring distance, with typical dimensions ranging from 25 cm to 100 cm. To reduce perspective distortion and improve measurement reliability, targets are generally installed at critical structural sections, while maintaining an observation configuration in which the camera line of sight is as close as possible to perpendicular to the target surface.

In addition, the target placement criterion considers parallax effects under oblique viewing angles. Preliminary tests were conducted by fixing the target on a rigid wall and varying the camera viewing angle from 0° to 60° in 5° increments using a pan-tilt platform. The results show that the projected target gradually changes from an approximately circular shape to an elliptical shape as the viewing angle increases. When the viewing angle exceeds about 20°, the difference between the major and minor axes becomes apparent, indicating increased parallax-related measurement errors. Therefore, to ensure measurement accuracy and stability, the viewing angle between the camera and the target surface should be kept within a small range, preferably not exceeding 20°, and a near-normal observation geometry is recommended in practical engineering deployment.

It should be noted that camera distortion correction is not explicitly included in the standard processing pipeline of this study. This is because the system primarily adopts medium- to long-focal-length lenses (e.g., 75 mm), for which lens distortion is relatively small and has a limited influence on displacement measurement accuracy within the region of interest. However, for applications involving short focal length or wide-angle lenses (e.g., 8–12 mm), distortion effects may become significant. In such cases, camera calibration procedures, such as Zhang’s method, can be employed to estimate the intrinsic and extrinsic parameters of the camera, and distortion correction can be applied prior to displacement extraction. This ensures the robustness and adaptability of the proposed method under different imaging configurations.

During monitoring, the system continuously acquires image sequences of the targets on the structural surface. The variation in the target center position is extracted through image feature recognition and subpixel localization techniques. Subsequently, the pixel coordinate changes are converted into actual displacement values using camera calibration relationships, thereby obtaining displacement time histories of the structure in both horizontal and vertical directions. By integrating displacement data from multiple monitoring targets, the overall deformation state of the structure can be characterized, providing a reliable data basis for structural condition assessment and health monitoring.

**Figure 4:**
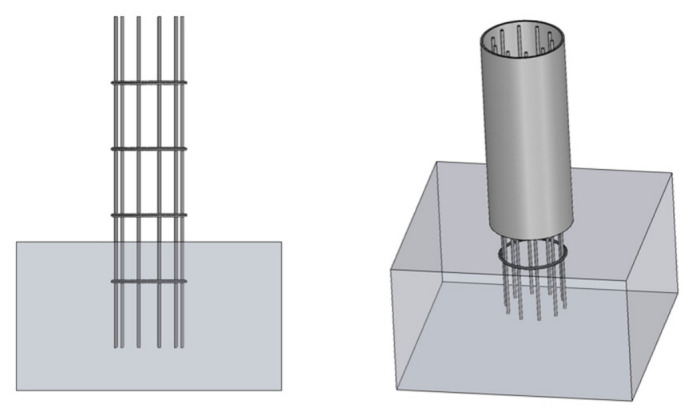
Installation of Equipment Observation Platforms.

As shown in Figure 4, to ensure sufficient mounting rigidity, the vision-based deformation sensor should be installed on a rigid concrete pedestal or a wall-mounted base and fixed using expansion bolts. For field deployment, a concrete foundation with a minimum size of 0.35 m × 0.35 m × 0.7 m (with at least 0.2 m above ground) is constructed, reinforced with a steel cage, and embedded to a depth not less than 0.5 m. In this study, C30-grade concrete is used to provide adequate stiffness. The sensor is installed after sufficient curing of the foundation.

For sites where excavation is not feasible, a grouted rebar anchoring method (depth ≥ 0.35 m) can be adopted. For wall-mounted installation, stainless steel expansion bolts (e.g., M8 × 70 mm) are used to fix the bracket and sensor.

During the initial commissioning period, installation stability is evaluated using the displacement of a fixed reference target. If a persistent displacement drift exceeding 2 mm is observed within the first 3 days, the mounting condition should be inspected and reinforced.

## 5. Application Case Study

### 5.1. Mid-Span Deflection Monitoring Results of the Bridge

**Figure 5:**
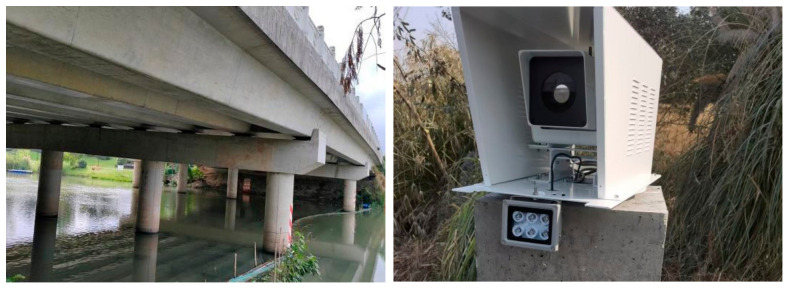
Test Setup.

**Figure 6:**
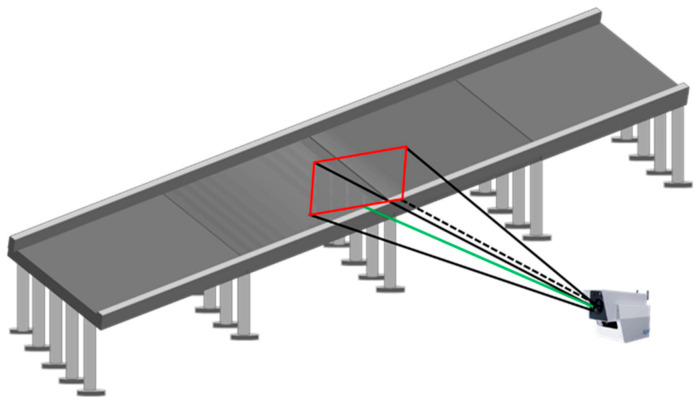
Schematic Diagram of the Monitoring Device.

To validate the capability of the proposed vision-based deformation monitoring system for detecting dynamic deflection in bridge field conditions, a 30-day continuous monitoring campaign was conducted on a girder bridge located in Yuhang District, Hangzhou, China (from 1 November 2025 to 30 November 2025), with a sampling interval of 10 min. A single embedded vision-based deformation sensor was employed to perform synchronous multi-point measurements of horizontal (x) and vertical (y) displacements at two monitoring points located at the mid-span. The field setup included the vision-based deformation sensor, two machine vision targets, a mobile storage unit, and a power supply system (the experimental layout is shown in Figure 5 and Figure 6).

**Figure 7:**
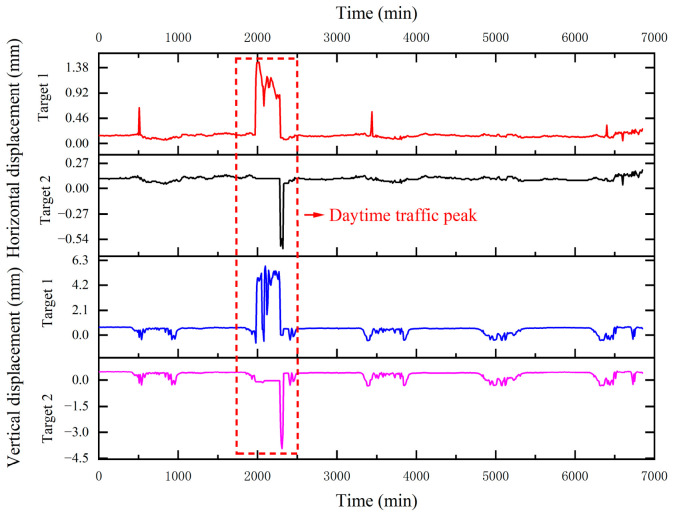
Displacement Curve of the Bridge Over a Week. I confirm.

The main findings from the time-series and statistical analyses of the raw data are summarized as follows. Overall, no significant long-term cumulative displacement or monotonic drift is observed during the monitoring period. The displacement time histories exhibit small-amplitude periodic fluctuations, indicating that the structure remained in a stable service state throughout the monitoring period. In terms of spatial direction, the displacement amplitude in the x-direction is relatively small and varies smoothly, predominantly within the sub-millimeter range. In contrast, the y-direction is more sensitive to external loads and exhibits more pronounced time-varying characteristics. During daytime traffic peak hours (approximately 09:00–13:00), the mean displacement in the y-direction shows a staged increase, which is consistent with the temporal distribution of traffic loading (see Figure 7).

**Figure 8:**
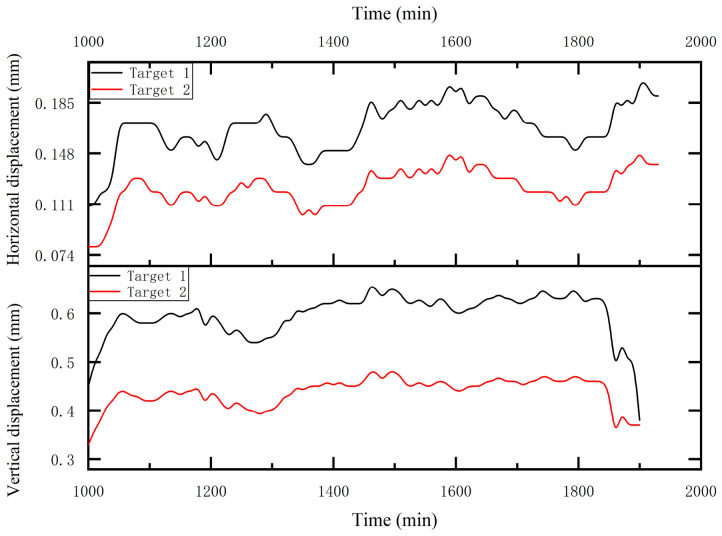
Displacement Curve During Periods of Stable Bridge Conditions.

To evaluate the resolution capability of the system under low-disturbance conditions, several “normal periods” with relatively weak traffic influence were selected for localized analysis. The displacement time series during these periods exhibit continuous and smooth small-amplitude fluctuations, without any abrupt jumps. Statistical results indicate that the displacement amplitudes during normal periods are mainly within the range of 0.1–0.2 mm, and the responses of multiple targets show a high degree of consistency, with only minor differences observed in local details (see Figure 8). The reference target remains stable around baseline level during the same period, indicating that the system background noise is well controlled and supporting the engineering reliability of the small displacement measurements obtained from the moving targets.

**Figure 9:**
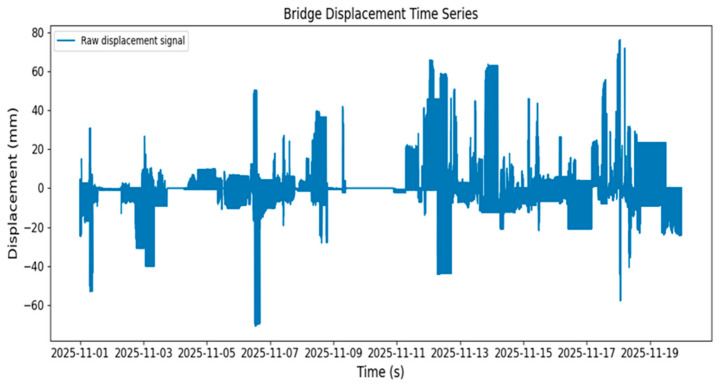
Time-series displacement signal obtained from the vision-based monitoring system during the observation period. Note: The plotted signal corresponds to displacement data after preprocessing and removal of unstable measurement segments.

The processed displacement dataset is further analyzed to evaluate the structural response of the bridge during the monitoring period. As shown in Figure 9, after removing a segment of unstable measurements caused by temporary visual tracking disturbances, the remaining data are used for statistical analysis.

**Figure 10:**
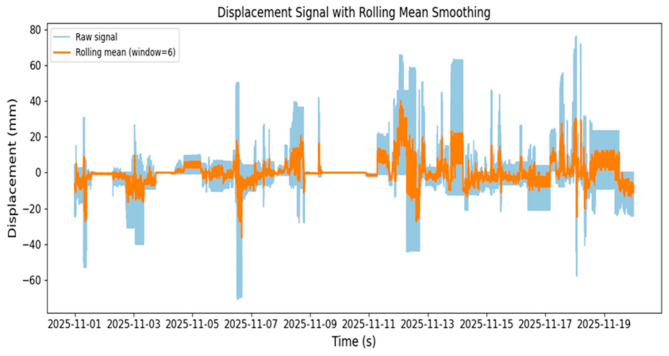
Rolling mean analysis of the displacement signal used to evaluate long-term measurement stability. Note: The plotted signal corresponds to displacement data after preprocessing and removal of unstable measurement segments.

As illustrated in Figure 10, the displacement signal remains generally centered around zero throughout the observation period. The calculated mean displacement is approximately 0.15 mm, while the standard deviation reaches 13.38 mm due to intermittent peak responses and transient disturbances contained within the full monitoring dataset. This indicates that the monitoring system maintained a stable reference baseline during the measurement period, without exhibiting significant long-term drift.

It should be noted that the relatively large standard deviation observed in the displacement signal is primarily influenced by transient peak responses and occasional disturbances associated with the vision-based tracking process. During normal monitoring periods, the displacement fluctuations remain within a much smaller range (approximately 0.1–0.2 mm), which is consistent with the expected structural behavior. The inclusion of intermittent peak events and measurement disturbances in the full dataset leads to an increased overall variance, but does not contradict the stability of the system under typical conditions.

**Figure 11:**
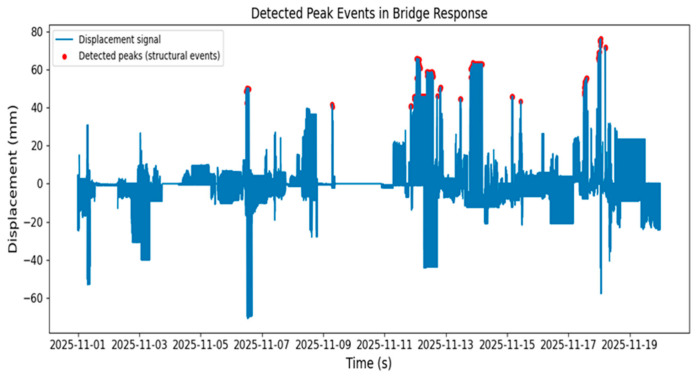
Peak displacement events detected using the ±(μ + 3σ) statistical threshold. Note: Both positive and negative displacement excursions exceeding the threshold are classified as peak events.

To identify transient displacement responses, a statistical peak detection method based on the μ + 3σ criterion is employed. As shown in Figure 11, a total of 259 peak events are detected in the dataset using this threshold. These peaks correspond to occasional large displacement responses in the time series, which may be associated with transient loading effects such as vehicle passages or temporary measurement disturbances during the vision-based monitoring process.

Overall, the time-series analysis indicates that the bridge displacement remains relatively stable under normal operating conditions, while intermittent peak responses are induced by short-term external influences. The statistical characterization of these displacement patterns provides additional quantitative insight into the structural behavior captured by the vision-based monitoring system.

### 5.2. Monitoring Results of the Sluice Structure

The sluice adopts an integral reinforced concrete chamber structure, with the foundation treated, using prestressed high-strength concrete pipe piles in soft soil conditions. The gate is a fish-belly-shaped double-arched spatial steel structure, and an open-box traffic bridge is arranged above the chamber. It is the largest sluice structure in the Qiantang River estuary region, which is characterized by strong tidal bores. Through the application of new deformation monitoring technologies, the safety monitoring system of the sluice is enhanced by strengthening capabilities in data acquisition, storage, processing, analysis, evaluation, and early warning, thereby enabling timely identification of potential safety risks and ensuring safe operation.

**Figure 12:**
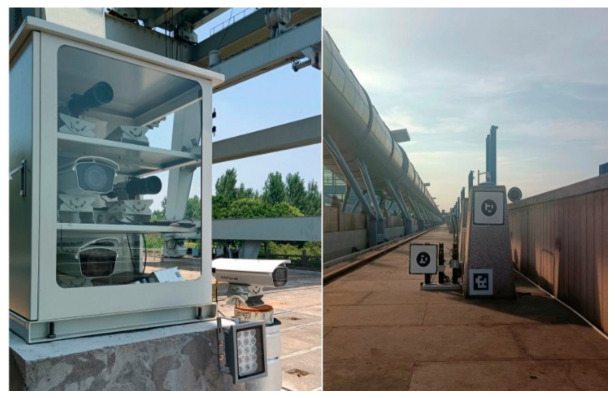
Layout of the Gate Test Facility.

In this project, ten vision-based deformation monitoring points are evenly arranged along the transverse centerline of the structure. Five vision-based deformation sensors are installed near the control buildings on both the left and right banks. As shown in Figure 12, one sensor located near the centerline, together with its corresponding targets, is selected for continuous displacement monitoring. In this test, the vision-based deformation sensor is installed directly on the structure. Since vision-based monitoring is susceptible to environmental disturbances such as illumination changes and slight camera motion induced by structural vibration, an additional fixed target is installed in a non-deforming region to provide a stable reference coordinate. The displacement of the fixed target is theoretically zero; any detected movement indicates potential system anomalies requiring data correction.

**Figure 13:**
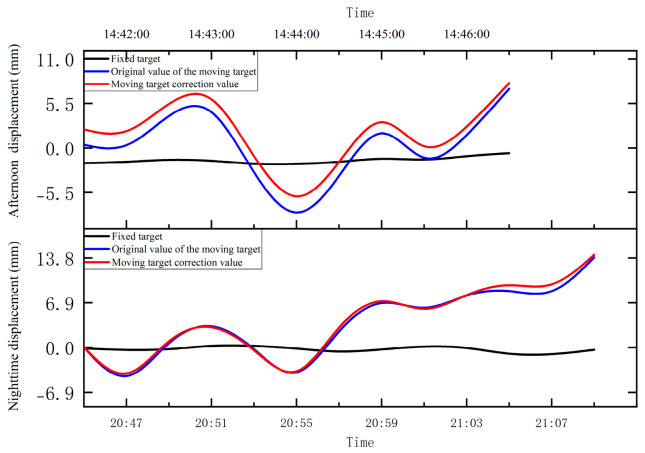
Vertical Displacement Curve of the Gate.

Short-term monitoring tests are conducted during the afternoon and nighttime under different illumination conditions on the same day, with sampling intervals of 30 s and 2 min, respectively. As shown in Figure 13, the displacement curves of the fixed target fluctuate slightly around zero in both tests without abrupt changes, indicating good short-term stability of the vision-based monitoring system. The sub-millimeter fluctuations observed in the fixed target also demonstrate the system’s sensitivity to small displacements. During the daytime, the displacement of the moving target shows a gradual increase from near zero to several millimeters, followed by a decrease. This slow and trend-like variation is consistent with the effects of tidal action or diurnal water-level fluctuations of the Qiantang River on the sluice structure. The nighttime test was conducted around 21:00; despite low illumination conditions, the displacement curve remains continuous and smooth, without significant noise amplification or signal interruption, demonstrating the robustness of the system under complex environmental conditions.

**Figure 14:**
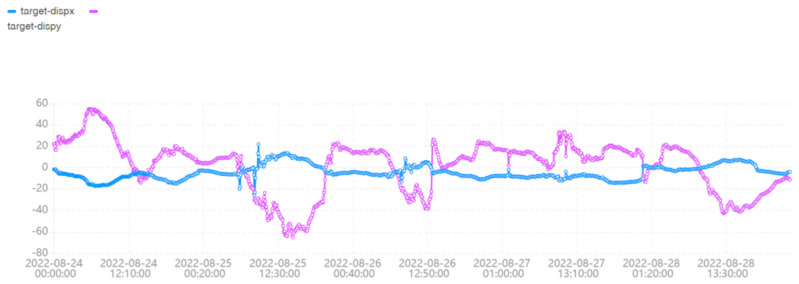
Displacement Curve of the Gate Over 5 Days.

To further evaluate the long-term performance of the vision-based monitoring system under tidal effects, a five-day continuous monitoring test is conducted. As shown in Figure 14, the system successfully captures significant displacement variations in the sluice during tidal cycles, with a maximum negative displacement of −51.9 mm and a maximum positive displacement of 54.7 mm. The continuous monitoring data reveal a strong correlation between sluice displacement and tidal processes. During the rising tide phase, the structure exhibits inward horizontal displacement accompanied by upward vertical movement, whereas during the falling tide phase, it shows outward horizontal displacement and downward settlement.

The strong correlation between the displacement response and tidal processes in the sluice case demonstrates that the vision-based monitoring system can accurately capture the deformation behavior of hydraulic structures under external hydrodynamic loading. Compared with the bridge case dominated by traffic loading, the displacement of the sluice structure exhibits more continuous and periodic characteristics, further validating the applicability of the proposed method to low-frequency, long-term deformation processes. Overall, the results indicate that the system can reliably capture relative displacement changes in the target object, with measurement errors within 1 mm under the given monitoring conditions, demonstrating its capability for long-term, high-precision structural health monitoring in environments subjected to periodic external forces such as tides.

## 6. Discussion

### 6.1. Reliability Analysis of Sub-Millimeter Monitoring Capability

The monitoring results from the bridge under low-disturbance periods and the low-frequency deformation of the sluice indicate that the proposed intelligent vision-based deformation monitoring method is capable of reliably detecting displacement changes on the order of 0.1 mm under field conditions. This capability is primarily attributed to the synergistic effects of several key techniques. First, the subpixel localization method based on surface fitting improves the quantization accuracy at the pixel level. Second, the filtering strategy combining Hilbert–Huang Transform (HHT) with predefined Gaussian white noise enhances the suppression of low-frequency platform disturbances, thereby improving the signal-to-noise ratio in practical field environments.

Compared with existing vision-based monitoring studies that typically achieve millimeter- or even centimeter-level displacement resolution through techniques such as template matching, feature tracking, digital image correlation, and multi-view geometric calibration, the field results presented in this study demonstrate that the proposed method can still effectively identify micro-displacements under long-term monitoring conditions, which is important for early-stage deformation detection in structural health monitoring [34].

These results help address the gap identified in the Introduction, namely, the lack of long-term field validation for micro-displacement monitoring under complex outdoor conditions.

### 6.2. Interpretation of the “Reverse Displacement” Phenomenon

In the bridge case, opposite-signed displacement responses in the same direction are occasionally observed at different targets. This phenomenon may be attributed to two possible causes.

On the one hand, from a structural mechanics perspective, under eccentric or non-uniform vehicle loading, local sections of the bridge may experience slight torsional responses, resulting in differences in displacement directions at different locations [35]. On the other hand, short-term environmental disturbances cannot be completely ruled out, such as temporary target occlusion, sudden illumination changes leading to feature point re-localization errors, or slight camera pose vibrations affecting image matching results [36].

These anomalies are typically instantaneous and non-persistent and do not substantially affect the overall displacement trend assessment. In engineering practice, these anomalies can be effectively identified and eliminated through multi-target cross-validation and constraints from fixed reference targets.

### 6.3. Limitations

Although the proposed method demonstrates good stability in field tests, several limitations should be noted. Vision-based techniques are sensitive to visibility and lighting conditions; heavy rain, dense fog, or strong backlighting may significantly reduce effective resolution. Long-distance targets may experience refractive distortions under high temperature gradients, affecting the stability of subpixel localization. In addition, the current method still requires manual verification or multi-source data fusion to distinguish between true structural responses and pseudo-signals under strong transient disturbances. The identified limitations are consistent with the research gap highlighted in the Introduction, where long-term stability and interference resistance under complex field conditions remain challenging for vision-based monitoring systems.

It should be noted that the current acquisition strategy is primarily designed for low-frequency and sustained structural deformation monitoring. Therefore, while the proposed system is effective for identifying quasi-static trends and sustained structural responses, short-duration high-frequency vibrations induced by fast-moving traffic may not be fully resolved under the present sampling configuration. This limitation does not affect the main objective of the present study, which focuses on long-term structural deformation monitoring, but it does indicate that future work should consider higher-frame-rate acquisition and adaptive multi-rate sampling to better capture transient traffic-induced responses.

Therefore, future work should focus on systematically integrating vision-based data with environmental sensors, as well as developing higher-frame-rate acquisition and adaptive multi-rate sampling strategies. Future work may also further integrate the proposed vision-based deformation monitoring framework with physics-informed or load-transfer-based modeling approaches to improve the interpretation of measured displacement responses in complex engineering systems [37,38]. Controlled step-displacement tests and outdoor comparative experiments should also be conducted to further quantify field accuracy and enhance the adaptability of the algorithms.

## 7. Conclusions and Prospects

This study develops a non-contact monitoring system integrating an embedded vision-based deformation sensor with intelligent algorithms and validates it in bridge and sluice engineering scenarios. Field results demonstrate that the system achieves sub-millimeter-level displacement measurement accuracy under typical monitoring conditions and maintains stable performance in long-term outdoor environments. It is capable of capturing both traffic-induced transient responses and tide-driven low-frequency structural deformations.

From a methodological perspective, the integration of high-precision optical imaging, surface-fitting-based subpixel localization, and a noise-assisted HHT-based filtering scheme enables robust extraction of structural displacement signals under environmental disturbances.

From an engineering validation perspective, the bridge monitoring results show no observable cumulative drift over a one-week period, indicating good long-term stability. In the sluice case, the measured displacement exhibits clear consistency with tidal variations, and the use of fixed targets confirms that environmental noise is effectively controlled. The proposed system supports flexible deployment and multi-point monitoring without extensive contact sensor installation, offering a cost-effective solution for structural health monitoring of large-scale infrastructure.

It should be noted that the method remains sensitive to adverse environmental conditions such as poor visibility, strong illumination variation, and severe atmospheric disturbances under long-distance imaging. In addition, the identification of certain transient anomalies may still require further validation or multi-source data support.

Future work will focus on improving environmental adaptability, integrating multi-source sensing, and extending the approach to three-dimensional vision-based monitoring for broader engineering applications.

## Data Availability

Data available on request due to restrictions.
